# Stress Exposure and Physical, Mental, and Behavioral Health among American Indian Adults with Type 2 Diabetes

**DOI:** 10.3390/ijerph14091074

**Published:** 2017-09-16

**Authors:** Melissa L. Walls, Kelley J. Sittner, Benjamin D. Aronson, Angie K. Forsberg, Les B. Whitbeck, Mustafa al’Absi

**Affiliations:** 1 Department of Family Medicine and Biobehavioral Health ,University of Minnesota Medical School, Duluth Campus, 1035 University Drive, 235 SMed, Duluth, MN 55812, USA; aforsber@d.umn.edu (A.F.); malabsi@d.umn.edu (M.A.); 2 Department of Sociology, Oklahoma State University, Stillwater, OK 74078, USA; kelley.sittner@okstate.edu; 3 Department of Pharmacy Practice, Ohio Northern University, Ada, OH 45810, USA; b-aronson.1@onu.edu; 4 Department of Sociology, University of Nebraska, Lincoln, NE 68588, USA; lwhitbeck2@unl.edu

**Keywords:** American Indian, diabetes, stress, Native American

## Abstract

American Indian (AI) communities experience disproportionate exposure to stressors and health inequities including type 2 diabetes. Yet, we know little about the role of psychosocial stressors for AI diabetes-related health outcomes. We investigated associations between a range of stressors and psychological, behavioral, and physical health for AIs with diabetes. This community-based participatory research with 5 AI tribes includes 192 AI adult type 2 diabetes patients recruited from clinical records at tribal clinics. Data are from computer-assisted interviews and medical charts. We found consistent bivariate relationships between chronic to discrete stressors and mental and behavioral health outcomes; several remained even after accounting for participant age, gender, and income. Fewer stressors were linked to physical health. We also document a dose–response relationship between stress accumulation and worse health. Findings underscore the importance of considering a broad range of stressors for comprehensive assessment of stress burden and diabetes. Policies and practices aimed at reducing stress exposure and promoting tools for stress management may be mechanisms for optimal health for AI diabetes patients.

## 1. Introduction

Stress process models of disease suggest that heightened exposure to stressors is associated with worse health outcomes, reduced capacity for disease management, and increased risk for disease-related complications and comorbidities [1,2]. Stress has been implicated in the etiology of type 2 diabetes mellitus for centuries [3], and stressors are known to compromise diabetes management, metabolic control, treatment compliance and quality of life [4,5]. Further, the stress process is influenced by social stratification systems whereby those occupying marginalized social and economic statuses are more likely to experience higher levels of stress [6].

American Indian (AI) communities simultaneously experience disproportionate levels of historical, social, and economic stressors and disparate rates of type 2 diabetes compared to the overall United States population [7]. Diabetes prevalence rates are double for AI and Alaska Native (AN) adults compared to the United States average (17.8% vs. 8.5%), representing the highest age-adjusted prevalence of any racial or ethnic group [8]. In 2013, diabetes was the 5th leading cause of death for AI/ANs of all ages and was a major contributor to the leading cause of death, cardiovascular disease [8]. AIs living with diabetes also experience significantly more comorbidities and complications like amputation, mental health disorders, and hypertension relative to the U.S. general population of adults with diabetes [9].

There has been substantial research on the origins and extent of the diabetes epidemic, its correlates, and complications among AI people [10,11,12,13]. Yet, there has been little empirical work on the connections between stress and diabetes outcomes for AIs [14]. In one exception, a study of two distinct tribal communities found that early-life trauma and neglect, environmental hassles, family dysfunction, community economic distress and discrimination were significantly higher among adults with diabetes than those without [5]. Notable limitations of that study included that these associations were not uniform across communities, the reliance on self-reported diabetes diagnosis, and overall impacts of stressors on diabetes-related outcomes were not examined.

Two important points regarding the empirical study of stress inform the current study. First, early efforts to detail the impact of stressors on health relied heavily on discrete measures like life events checklists [15]. Over time, critiques of the validity of checklist approaches propelled researchers to consider the vast “realm of stressors” [16] and more adequately operationalize potential stressful experiences (6). To conceptualize this approach, Wheaton’s [16,17] stress continuum promotes consideration of a range of stressful experiences and typologies. At one end, acute stressful events with discrete beginning and end points provide an anchor, with more chronic stressors (i.e., enduring, often open-ended) situated at the opposite end of the spectrum. Thus, this continuum considers impacts of stress exposure across a spectrum of stressor typologies and domains.

The second critical consideration lies in exclusion of diverse perspectives in the formation of stress measurement, particularly those stressors most salient to racial/ethnic minority and marginalized groups [18]. Studies relying solely on commonly used stress measures may thus underestimate the impact of stressors on health and health behaviors. The sources of stress and consequences in AI communities in particular are poorly understood in terms of the range and meaning of stressors experienced [19]. To this end, our approach to measuring stress in American Indian communities for the current study was heavily informed by qualitative community feedback (see Section 2).

The purpose of this study is to investigate linkages between a continuum of stressors and diabetes-related health factors among AI adults living with type 2 diabetes. Specifically, we examine the direct, cumulative and relative associations between stressors and behavioral, mental, and physical health outcomes. Our selection of stressor variables was driven by our goals to (a) incorporate a continuum of stressor possibilities ranging from discrete (e.g., life events) to chronic (e.g., distress related to chronic disease) and (b) be responsive to tribal community members’ identification (via focus groups) of stressors most salient to AIs living with type 2 diabetes [20]. In accord with previous research, we expect that (Hypothesis 1) higher reports of stressor exposure will be significantly associated with poorer health, and (Hypothesis 2) a “dose–response” relationship will occur between stressor accumulation and health whereby progressively worse health outcomes are observed as number of stressors reported increases. Further, we explore the relative associations between individual stressors and health when accounting for demographic factors and a range of other stressors.

## 2. Materials and Methods

Data for this study are from the Maawaji’ idi-oog Mino-ayaawin (Gathering for Health) project, a longitudinal community-based participatory research (CBPR) collaboration between the University of Minnesota Medical School, Duluth campus and five Ojibwe communities in Minnesota and Wisconsin. Community Research Councils (CRCs) comprised of an average of 6 members on each reservation are active partners in the entire research process and have participated in all aspects of study planning, protocol development, and implementation to ensure cultural and local acceptability of study procedures. CRC members also serve as co-authors and co-presenters on various data dissemination activities and ongoing work to translate findings into locally relevant programming and services. Clinic-based staff members in each community assisted the team with participant selection and medical chart reviews. Final protocol for the study was reviewed and approved by the University of Minnesota Institutional Review Board (Study #1206S16361) and the National Indian Health Service Institutional Review Board, and this manuscript was reviewed by all CRC members prior to journal submission.

### 2.1. Sample

Clinical staff at each tribal site’s medical facility generated simple random samples for study recruitment using clinic patient records. Inclusion criteria were a diagnosis of diabetes documented in the medical record within 5 years of the sampling date, age 18 years or older, and self-identified as American Indian. A total of 194 participants enrolled in the study, representing a baseline response rate of 67%. Data for this report include responses from the 192 participants who completed a baseline survey interview between November 2013 and November 2015.

### 2.2. Procedure

The survey for this study was heavily informed by community feedback derived from two sets of focus groups at each of the five participating tribal sites. In the first groups, community members were queried about sources of stress common in their tribal community; thematic results from Group 1 were used to identify and/or adapt stress measures that were presented to participants in Group 2 for feedback on validity, comprehensiveness, and understandability.

For the survey component of the study, clinic staff sent study invitation letters and brochures to residences of randomly selected patients. Non-refusing individuals were contacted by trained community interviewers, screened for study eligibility, and formally invited to participate. Visits were scheduled at a location of participants’ choosing, at which time interviewers gathered signed informed consent and HIPAA authorization forms. Data from interviewer computers were electronically synced via Internet connection to a secure server at the university, uploaded, and converted to SPSS data files. Data for this manuscript include responses to baseline Computer-Assisted Personal Interviews (CAPI) and clinical chart reviews, for which participants received a $50 incentive and a small, culturally meaningful gift. Clinical staff completed medical chart reviews using data from patient health records.

### 2.3. Measures

Three control variables are included in these analyses: *age* (in years), *gender* (male = 0, female = 1), and *per capita household income* were each assessed via self-report survey responses. Five dependent variables are used: two physical health, one mental health, and two behavioral. One physical health outcome, *hemoglobin A1c* (*HbA1c*), was retrieved from patient medical charts. Clinical staff recorded the most recent HbA1c value available for each participant. Project interviewers trained in proper anthropometric measurement and recording techniques collected an additional measure of physical health, *waist-to-hip ratio*. Waist-to-hip ratios are widely used as a measure of health status and risk for several diabetes-related comorbidities including cardiovascular events [21]. All remaining outcomes were assessed via survey responses. As an indicator of mental health status, *depressive symptoms* were measured using continuous responses to the Patient Health Questionnaire (PHQ-9) [22] with a possible range of 0–27 and higher values representing more depressive symptoms. *Medication adherence* questions were asked to a subsample of 166 participants indicating that they used oral or injectable diabetes medications and were measured using the 4-item Morisky Medication Adherence Scale (MMAS-4) [23]. Items from the MMAS-4 were summed, creating a possible score ranging from 0 to 4, with higher scores indicating higher adherence. Average responses to two items from the Summary of Diabetes Self-Care Activities (SDSCA) diet subscale was used to assess days/week *adherence to a healthy diet plan* (possible range = 0–7 days) [24,25].

**Figure 1:**
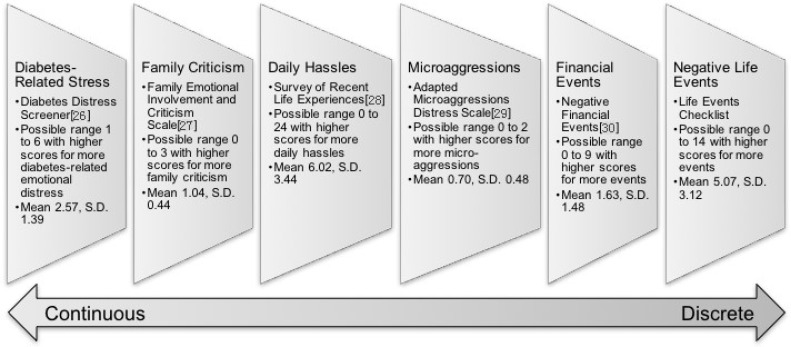
Stress measurement sources, scoring, means, standard deviations (S.D.), and position on a continuum.

Six measures of psychosocial stressor exposure were included in these analyses; the sources, means, standard deviations, and brief descriptions of measurement properties are described in Figure 1 [26,27,28,29,30]. Each stressor is mapped onto an adaptation of Wheaton’s continuum [16,17] ranging from stressors more chronic/continuous in their operationalization on the left to those more discrete/acute on the far right. *Diabetes-Related Stress (Diabetes Distress)* assesses feelings of being overwhelmed by diabetes management. *Family Criticism* items gauge perceptions of family disapproval and discouragement. *Daily Hassles* are indicated by everyday difficulties and nuisances such as problems with work, vehicles, technology, and home repairs. *Microaggressions* represent a range of racial insults including subtle but pervasive experiences like feelings of invisibility, portrayal of Native people as mascots, and hearing that one doesn’t “look” Indian. *Negative Financial Events* assess discrete adverse financial experiences occurring in the 6 months prior to interview date (e.g., being laid off or becoming unemployed, losing benefits, repossession, etc.). *Stressful Live Events* are indicated by a checklist of possible stressful experiences such as moving, job changes, and role transitions. Participants were asked to consider stressors experienced within the 6 months prior to the interview for all of the scales with the exception of daily hassles, which was limited to the past month. Using each of these stressor variables, we also calculated a cumulative score of above average stress exposure. We recoded each of the 6 stressors so that individuals scoring above the mean for a given stress variable = 1, and those scoring at or below the mean = 0. We then created a summation of above-average stressors that revealed substantial outliers; namely, n = 9 participants scoring 5 and n = 13 scoring 6; thus, we collapsed these two response categories for a final variable ranging in value from 0 to 4 or more stressors.

### 2.4. Analytic Approach

We generated Pearson’s correlation coefficients to describe focal relationships between individual stressors and health outcomes. Means comparisons were used to determine the associations between accumulation of above-average stressors and health. Lastly, we included all stressor variables in a single ordinary least squares regression analysis for each health outcome to identify the relative influence of stressors. Because of possible statistical power limitations and intercorrelations among some of the stressors, exact *p*-values are displayed alongside multivariate results and alpha was set at *p* < 0.10.

## 3. Results

A little over one-half (56%) of the study participants were female, with a mean age of 46.3 years (SD = 12.21). Average per capita household income was $9767 (SD = 8901). The sample mean HbA1c was 7.7% (61.1 mmol/mol) (SD 2.2%; SD 24.2 mmol/mol), the average waist-to-hip ratio was 1.03 (SD = 0.29), and the mean PHQ-9 depression score was 5.27 (SD = 5.6). The average medication adherence score was 2.53 (SD = 1.3), and participants reported following a healthy diet plan an average of 2.9 days per week (SD = 1.6).

Bivariate relationships between individual stressors and outcomes are displayed in Table 1. HbA1c levels were positively and significantly associated with diabetes distress. Two stressors, negative financial events, and negative life events were related to larger waist-to-hip ratios. All six of the stressors were significantly and positively correlated with depressive symptoms; the strength of the correlation was strongest for family stress followed closely by microaggressions and daily hassles. Five of the stressors included in this study were significantly related to worse medication adherence with the largest correlation coefficient observed for diabetes distress. Four stressors were linked to worse diet adherence: diabetes distress, family criticism, daily hassles, and negative financial events; the largest correlation coefficient of these was financial events. 

**Figure 2:**
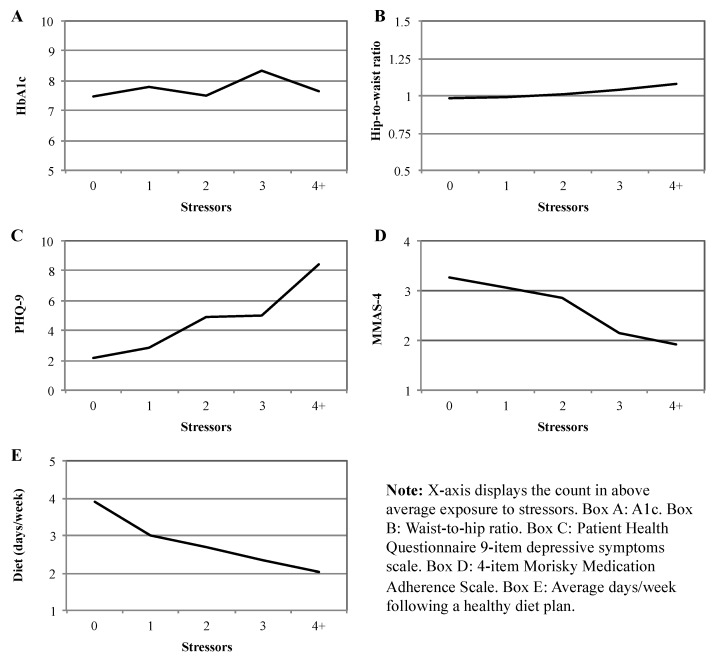
Accumulation of stressors by health outcomes.

Our next step was to investigate the potential cumulative impact of stressor exposure on health behaviors and outcomes. Means comparisons results are displayed in Figure 2, with mean values for each health outcome displayed on the y-axis and the cumulative above-average stressor values plotted across the x-axis. With the exception of HbA1c, exposure to above-average stressors accumulation was paired with worsening health/health behaviors. These trends were statistically significant for depressive symptoms (*F* (4, 184) = 9.23; *p* < 0.001), medication adherence (*F* (4, 184) = 8.82; *p* < 0.001), and diet plan adherence (*F* (4, 184) = 3.03; *p* < 0.05).

Our final set of analyses explore the relative influence of individual stressors on health when taking into account possible effects of gender, age, per capita household income, *and* exposure levels to all other stressors on the continuum. Looking first at the control variables, older participants had significantly lower HbA1c values and better medication and diet plan adherence. Females had significantly lower waist-to-hip ratios and higher depressive symptoms than did males. Per capita household income was unrelated to any of the outcomes. In Model 1, after accounting for controls and other stressors, diabetes distress remained a significant predictor of higher HbA1c levels. Those reporting more negative financial events had greater waist-to-hip ratios in Model 2. Both family criticism and microaggressions were positively and significantly related to depressive symptoms (Model 3) and had similar effect sizes (i.e., standardized coefficients). Diabetes distress and daily hassles were negatively associated with medication adherence with comparable effect sizes as shown in Model 4. In Model 5, diabetes distress and financial events were negatively associated with adherence to a diet plan, again with similar effect sizes observed.

## 4. Discussion

Type 2 diabetes mellitus has been labeled an epidemic in many AI communities and has motivated research to promote a deeper understanding of processes influencing diabetes outcomes for AI people. While stressors have long been known to influence the onset and course of diabetes, we move beyond existing literature to investigate associations between a continuum of stressors [16,17] and health outcomes within five AI communities with consideration of family, chronic disease, financial, and minority (microaggressions) stress contexts. 

In support of Hypothesis 1, our findings reveal linkages between stress exposure and worse behavioral, psychological, and physical health for AI people. A majority of the stressors from across the continuum (Figure 1) were associated with worse behavioral (medication and diet plan adherence) and mental (distress) health in our bivariate models. Diabetes distress was significantly related to poorer physical health in terms of higher HbA1c values. In addition, the two most discrete measures stressors, negative financial and stressful life events, were positively associated with waist-to-hip ratios in bivariate analyses. Thus, while we found at least one significant association between stress and every type of outcome in this study, the stressors were most consistently correlated with worse behavioral and mental health conditions (as opposed to physical health). Depression and nonadherence to medication and diet regimes have been linked to poorer diabetes-related health including microvascular and macrovascular complications, hospitalizations, and Emergency Department visits [31,32,33], so it is possible that the influences of stressful experiences on mental and behavioral health may lead to worse physical health later on.

In partial support of Hypothesis 2, exposure to multiple stressors appeared to have an accumulating, negative effect that was statistically significant for diet plan adherence, medication adherence, and depressive symptoms (Figure 2). Thus, those participants reporting the highest levels of stress across the continuum were most at risk for treatment non-adherence and depression. Because most of the stressors in this study focus on recent experiences, the accumulation of life-course adversity by way of earlier or childhood stress exposure is not captured in these analyses and warrants future study.

Our community-informed process of stress measurement and the culturally specific focus of the study is an important contribution given historical omission of Indigenous voices in stress measurement. Several of our findings are consistent with previous research in samples not including AI participants. For instance, our general conclusion that stress is associated with worse diabetes-related outcomes is on par with prior work in diverse settings, as is the finding that women exhibited higher levels of depressive symptoms than did men [34,35]. Still, the cultural context of our study allows for a deeper interpretation of general findings. For instance, being older was associated with lower HbA1c levels and better medication and diet plan adherence. From an Indigenous perspective, this “elder effect” bolsters community calls for intergenerational programming and cultural reverence for elders, suggesting their important role in diabetes prevention, intervention, and education within AI communities. As another example, microaggressions represent subtle, everyday forms of discrimination [36] and were significantly correlated with depressive symptoms in this sample. Our measure directly specified microaggressive experiences on the basis of AI group membership, and this points to the importance of culturally safe care and consideration of privilege dynamics in diabetes treatment for non-majority cultural group members. This may be particularly critical for AI communities where legacies of medical mistreatment are coupled with some of the highest rates of type 2 diabetes in the nation. Family stress is an arguably ubiquitous form of stress cross-culturally, but the close-knit nature of the small, rural AI communities included in this study, the relevance of large extended family kinship networks, and the Indigenous cultural notions of belongingness may create special salience and/or impact of family-related stressors for health [37].

While many researchers consider diabetes distress as an outcome of living with type 2 diabetes, we conceptualized it here as a disease-specific stressor and an indication of patient struggles with diabetes management. The results in Table 1 show that diabetes distress was associated with all outcomes with the exception of waist-to-hip ratio. Further, the bivariate correlation between diabetes distress and depressive symptoms was modest in strength (*r* = 0.20; *p* < 0.01). These findings are in line with the work of Fisher and colleagues who have distinguished diabetes-specific distress from psychopathology, thereby highlighting the need for consideration of contextual influences on distress for diabetes patients [38]. Other studies have shown that diabetes distress is associated with poorer glycemic control and worse diabetes outcomes [33,39]; however, these studies have not included other stressors. In the present study, diabetes distress was significantly related to HbA1c, medication adherence, and diet plan adherence net the effects of other stressors and control variables.

Survey-based health research has historically focused primarily on discrete forms of stress (e.g., stressful life event checklists). Our examination of the relative influence of stressors vis-à-vis one another (Table 2) showed that chronic/continuous strains more frequently remained significant predictors of diabetes outcomes than did discrete forms of stress; in fact, negative life events (perhaps the most discrete form of stress in this study) were no longer associated with health outcomes in these models. Previous authors have argued that enduring, chronic stress may be more likely to generate allostatic load (i.e., wear and tear on the body that can amass with repeated exposure to stress) and therefore create vulnerability to disease and health problems [40], and our findings fall in line with this argument.

We recognize some important limitations exist in this investigation including the cross-sectional data, which yield findings that are correlational rather than causal in nature. Because of the multitude of significant bivariate associations among the independent variables and a limited sample size, the multivariate results shown in Table 2 may be underpowered for detecting meaningful associations between individual stressors and outcomes relative to all other stressors in the model. We thus chose 0.10 as an alpha value for determining statistical significance which increases possibilities of type 1 errors (i.e., false positives). Our attempt to map stressors onto a continuum ranging from discrete to chronic in nature is an imperfect approximation of lived experience. For example, while we measure financial events as singular instances with clear beginning and end points and thus position this measure on the discrete end of the continuum, such events may signal chronic experiences with financial strain. In addition, our measure of stressor accumulation does not account for possible differential effects (e.g., weighting) of specific stress measures. These conceptual limitations should be noted when interpreting findings.

## 5. Conclusions

The results of this study suggest that consideration of a range of stressor typologies is necessary for a complete understanding of processes influencing mental, behavioral, and physical health for AI patients living with type 2 diabetes. A logical next step is to identify coping resources and responses that may mitigate or offset the negative impacts of stress on health. In addition, longitudinal analyses will permit examination of stress proliferation, or the processes through which primary stressors give rise to additional stressful experiences [41]. For example, pervasive chronic strains like poverty may contribute to more discrete/acute stressful experiences. Of further importance is identifying how we can intervene on the proliferation process to reduce stress burden for patients living with chronic diseases. From a clinical standpoint and given these connections between stress and diabetes health, in-depth social histories may help diabetes care providers understand patient experiences with stress in order to activate coping tools and open doors for coping resources. In addition, policies aimed at ameliorating chronic exposure to stressors like financial strain (poverty) and educational programing to increase awareness of the nature and impact of microaggressive experiences may address root determinants of stress exposure for AI patients.

## Figures and Tables

**Table 1:** Pearson’s correlation coefficients for major study variables.

	1	2	3	4	5	6	7	8	9	10	11
1. Diabetes Distress	1										
2. Family Criticism	0.11										
3. Daily Hassles	0.44 ***	0.23 **	1								
4. Microaggressions	0.22 **	0.01	0.14 *	1							
5. Financial Events	0.15 *	0.12	0.17 *	0.21 **	1						
6. Negative Life Events	0.16 *	0.06	0.13	0.43 ***	0.38 ***	1					
7. HbA1c	0.18 *	0.12	0.04	−0.04	−0.04	−0.03	1				
8. Waist-to-Hip Ratio	0.12	0.08	0.07	0.09	0.21 **	0.15 *	−0.04	1			
9. Depressive Symptoms	0.20 **	0.28 ***	0.26 ***	0.27 ***	0.20 **	0.22 **	0.03	−0.01	1		
10. Medication Adherence	−0.31 ***	−0.16 *	−0.30 ***	−0.14	−0.22 **	−0.18 *	−0.04	−0.01	−0.29 ***	1	
11. Adherence to Diet Plan	−0.25 ***	−0.20 **	−0.23 ***	−0.03	−0.27 ***	−0.13	−0.08	−0.12	−0.24 ***	0.34 ***	1

Notes: *** Correlation is significant at the 0.001 level (2-tailed). ** Correlation is significant at the 0.01 level (2-tailed). * Correlation is significant at the 0.05 level (2-tailed).

**Table 2:** Ordinary least squares regression analyses of the relative relationships between stressors and health outcomes.

	Model 1				Model 2			Model 3			Model 4			Model 5	
	HbA1c			Waist-to-Hip Ratio			Depressive Symptoms			Medication Adherence			Adherence to Diet Plan		
	B	β	*p* Value	B	β	*p* value	B	β	*p* Value	B	β	*p* Value	B	β	*p* Value
	(SD)			(SD)			(SD)			(SD)			(SD)		
(Constant)	9.23		0.00	0.96		0.00	−0.71		0.73	2.73		0.00	2.88		0.00
Age (years)	−0.04	**−0.21**	0.01	−0.00	−0.05	0.50	−0.03	−0.07	0.29	0.02	**0.24**	0.00	0.02	**0.18**	0.01
	(0.01)			(0.00)			(0.03)			(0.01)			(0.01)		
Gender (Female = 1)	−0.52	−0.12	0.12	−0.08	**−0.14**	0.07	2.26	**0.20**	0.00	−0.31	−0.12	0.10	0.32	0.10	0.16
	(0.33)			(0.04)			(0.77)			(0.18)			(0.22)		
Household Income	−0.02	−0.06	0.40	0.00	0.04	0.65	−0.06	−0.09	0.18	0.01	0.04	0.62	0.02	0.12	0.10
	(0.02)			(0.00)			(0.04)			(0.01)			(0.01)		
Diabetes Distress	0.36	**0.22**	0.01	0.02	0.11	0.21	0.00	0.00	1.00	−0.14	**−0.15**	0.08	−0.19	**−0.16**	0.03
	(0.13)			(0.02)			(0.03)			(0.08)			(0.09)		
Family Criticism	0.45	0.09	0.23	0.02	0.03	0.68	3.07	**0.24**	0.00	−0.22	−0.08	0.28	−0.40	−0.11	0.11
	(0.37)			(0.05)			(0.86)			(0.20)			(0.25)		
Daily Hassles	−0.02	−0.04	0.65	0.00	0.02	0.85	0.17	0.10	0.17	−0.05	**−0.14**	0.09	−0.06	−0.12	0.12
	(0.05)			(0.01)			(0.12)			(0.03)			(0.04)		
Microaggressions	−0.22	−0.05	0.55	0.00	0.00	1.00	2.62	**0.23**	0.00	−0.09	−0.04	0.65	0.20	0.06	0.42
	(0.37)			(0.05)			(0.87)			(0.21)			(0.25)		
Financial Events	−0.16	−0.11	0.18	0.03	**0.15**	0.07	0.20	0.05	0.48	−0.06	−0.07	0.36	−0.18	**−0.17**	0.03
	(0.12)			(0.02)			(0.28)			(0.07)			(0.08)		
Negative Life Events	−0.01	−0.02	0.83	0.01	0.06	0.46	0.12	0.07	0.39	−0.03	−0.07	0.38	0.00	−0.01	0.92
	(0.06)			(0.01)			(0.14)			(0.03)			(0.04)		

Note: Two-tailed tests of significance. Bold indicates those coefficients that are statistically significant (*p* < 0.10).
